# Direct therapeutic targeting of immune checkpoint PD-1 in pancreatic cancer

**DOI:** 10.1038/s41416-018-0298-0

**Published:** 2018-10-31

**Authors:** Mei Gao, Miranda Lin, Richard A. Moffitt, Marcela A. Salazar, Jinha Park, Jeffrey Vacirca, Chuan Huang, Kenneth R. Shroyer, Minsig Choi, Georgios V. Georgakis, Aaron R. Sasson, Mark A. Talamini, Joseph Kim

**Affiliations:** 1 Department of Surgery, University of Kentucky, Lexington, KY USA; 2 Markey Cancer Center, University of Kentucky, Lexington, KY USA; 3 Department of Pathology, State University of New York, Stony Brook, NY USA; 4 Department of Experimental Therapeutics, City of Hope, Duarte, CA USA; 5 Department of Radiology, University of Iowa, Iowa City, IA USA; 6 New York Cancer Specialists, East Setauket, New York, NY USA; 7 Departments of Radiology, State University of New York, Stony Brook, NY USA; 8 Departments of Psychiatry, State University of New York, Stony Brook, NY USA; 9 Departments of Medicine, State University of New York, Stony Brook, NY USA; 10 Departments of Surgery, State University of New York, Stony Brook, NY USA

**Keywords:** Pancreatic cancer, Preclinical research

## Abstract

**Background:**

Pancreatic cancer (PC) hijacks innate cellular processes to promote cancer growth. We hypothesized that PC exploits PD-1/PD-L1 not only to avoid immune responses, but to directly enhance growth. We also hypothesized that immune checkpoint inhibitors (ICIs) have direct cytotoxicity in PC. We sought to elucidate therapeutic targeting of PD-1/PD-L1.

**Methods:**

PD-1 was assessed in PC cells, patient-derived organoids (PDOs), and clinical tissues. Then, PC cells were exposed to PD-L1 to evaluate proliferation. To test PD-1/PD-L1 signaling, cells were exposed to PD-L1 and MAPK was examined. Radio-immunoconjugates with anti-PD-1 drugs were developed to test uptake in patient-derived tumor xenografts (PDTXs). Next, PD-1 function was assessed by xenografting *PD-1*-knockdown cells. Finally, PC models were exposed to ICIs.

**Results:**

PD-1 expression was demonstrated in PCs. PD-L1 exposure increased proliferation and activated MAPK. Imaging PDTXs revealed uptake of radio-immunoconjugates. *PD-1* knockdown in vivo revealed 67% smaller volumes than controls. Finally, ICI treatment of both PDOs/PDTXs demonstrated cytotoxicity and anti-MEK1/2 combined with anti-PD-1 drugs produced highest cytotoxicity in PDOs/PDTXs.

**Conclusions:**

Our data reveal PCs innately express PD-1 and activate druggable oncogenic pathways supporting PDAC growth. Strategies directly targeting PC with novel ICI regimens may work with adaptive immune responses for optimal cytotoxicity.

## Introduction

The continuing rise in the incidence of pancreatic ductal adenocarcinoma (PDAC) will soon make it the second leading cause of cancer-related deaths in the US.^1^ The etiologies that underlie this deadly phenotype are many and one important mechanism may include the hijacking of innate cellular functions to support PDAC growth. For example, several studies including our own prior investigations have revealed that chemokine receptors expressed on PDAC cells enhance growth and invasiveness, which is in contrast to their innate functions orchestrating cell migration during organogenesis and inflammatory responses.^2–5^ PDAC, along with many other cancers, also employs the inhibitory effects of immune checkpoints to evade death from cytotoxic T-cell activity. In this paradigm, programmed death-ligand 1 (PD-L1) on PDAC cells interacts and binds to programmed cell death protein 1 (PD-1) on immune cells, thus blocking cytotoxic adaptive immune responses.^6^ However, based on our experience with PDAC and its exploitation of chemokine receptor functions, we hypothesized that PDAC cells also misappropriate immune checkpoints to directly enhance growth and invasiveness. The testing of our hypothesis may support a paradigm shift on the primary role and function of PD-1 and PD-L1 on PDAC cells.

There have been great interest and promise regarding the therapeutic potential of immune checkpoint inhibitors (ICIs) for a variety of cancers. These ICIs include monoclonal antibodies that target PD-1 or PD-L1 and block their interactions, thus releasing the inhibitory holds placed on cytotoxic T-cell activity and enabling adaptive immune responses to occur. Thus, consequent to our primary hypothesis that PDAC cells exploit immune checkpoints to enhance cancer growth, we also hypothesized that ICIs have direct cytotoxic effects on PDAC cells, independent of cytotoxic immune responses.

To test our two major hypotheses, we utilized human PDAC cells and tissues, and queried established PDAC datasets.^7–9^ Our PDAC models included cancer cell lines, patient-derived organoids (PDOs), and patient-derived tumor xenografts (PDTXs). All of these models are deficient in immune cell populations,^10^ and facilitate the investigation of autonomous immune checkpoint function and direct cytotoxic effects of ICIs in PDAC cells and tissues. Here, our studies revealed that PDACs innately express both PD-1 and PD-L1, suggesting that these immune checkpoints function beyond imparting immune tolerance alone. Exposure of PD-L1 to cultured PDAC cells revealed enhanced proliferation and novel activation of oncogenic signaling pathways. Importantly, our results showed that current clinical ICIs are directly cytotoxic to PDACs in vitro and in vivo and yielded higher cytotoxicity when combined with inhibitors of PD-1-mediated signaling pathways. Altogether, our studies provide novel understanding of PD-1 enhanced growth in PDACs and highlight the clinical prospects of increasing ICI efficacy with rationally designed drug regimens.

## Materials and methods

### Reagents

Human recombinant PD-L1 (Peprotech), nivolumab (anti-PD-1 monoclonal antibody, Bristol Myers Squibb), pembrolizumab (anti-PD-1 monoclonal antibody, Merck), atezolizumab (anti-PD-L1 monoclonal antibody, Genentech), trastuzumab (anti-HER2 monoclonal antibody, Genentech), daratumumab (anti-CD38 monoclonal antibody, Janssen), and trametinib (anti-MEK1/2 small molecule, Novartis) were obtained. Treatment assays with PD-L1 were performed at a concentration of 1 µg/ml as described.^11^ The following antibodies were used for western blot assay and immunofluorescence (IF): anti-PD-1 (Proteintech), anti-PD-L1 (Abcam), anti-phospho and total ERK (Cell Signaling), anti-GAPDH (Santa Cruz), and anti-beta-actin (Sigma Aldrich). PD1 (PDCD1) (NM_005018) Human Overexpression Lysate (Origene) and empty vector cell lysate (Origene) were used as PD-1 positive and negative controls, respectively.

### Cell line and organoid culture

Human pancreatic cancer cell lines MIAPaCa-2 and PANC-1 were obtained from ATCC. Cells were cryopreserved in liquid nitrogen upon receipt. Cells were maintained as previously described.^12,13^ The human pancreatic duct epithelial cell line (H6c7) was purchased (Kerafast) and cultured in Keratinocyte SFM with epidermal growth factor and bovine pituitary extract (Invitrogen) supplemented with 1× antibiotic–antimycotic (Gibco). Human Jurkat cells (gift from Dr. Jingfang Ju, Stony Brook University) was cultured in RPMI-1640 (Fisher Scientific) with 10% FBS and 1% P/S; and was used as PD-1 positive cell line control.

PDAC PDOs were maintained in standard fashion. L cells that produce Wnt3A (gift from Dr. Hans Clevers, Hubrecht Institute) were cultured in DMEM with 10% FBS and 1% P/S.^14^ 293T cells that produce Rspo1 (Trevigen) were cultured in DMEM with 10% FBS and 1% P/S and changed to Advanced DMEM/F12 with 10% FBS and 1% P/S before collecting conditioning medium.^15^ Activities of Wnt3A and Rspo1 were assessed by TopFlash assay using Dual-Glo Assay System (Promega).

### Immunoblotting and IF of cell lines and organoids

Western blot assay and IF were performed for proteins of interest. For IF, PDAC cells were seeded in 24-well plates and allowed to attach overnight. Cells were fixed, permeabilised, and blocked with bovine serum albumin (BSA). We probed with anti-PD-1 and anti-PD-L1 antibodies (1:100 dilution) with incubation overnight at 4 °C. Alexa fluor 555-tagged secondary antibodies (ThermoFisher) were then used for detection. Secondary antibodies alone were used as negative controls. DAPI solution was used for nuclear counterstaining and cell staining was photographed by inverted microscopy (Nikon Eclipse Ti–S).

For PDO staining, organoids were seeded (20 µl) in eight-well chamber slides (Lab-Tek). On days 3–4 following plating, medium was removed and PDOs were washed with phosphate-buffered saline (PBS) and fixed (2% paraformaldehyde). After washing with 1× PBS/glycine washing buffer ×3, wells were blocked with 1× IF wash buffer containing 10% horse serum for 2 h. Primary antibodies to verify pancreatic epithelial origin of tissues anti-K19 (1:10, TROMA-III from the University of Iowa) and anti-Sox9 (1:100, Millipore) were incubated overnight, and then secondary fluorochrome-labeled antibodies were used. DAPI solution was used for nuclear staining and slides were visualized by confocal microscopy (Zeiss 510 Meta NLO). PD-1 and PD-L1 staining for PDOs was performed in 24-well plates using the same methods as the cell lines.

### Human pancreatic cancer datasets

Normalized mRNA expression and associated metadata data were obtained and assessed from the Broad Institute, The Cancer Genome Atlas (TCGA) study of PDAC, and GSE71729 as previously described.^7–9^

### Cell proliferation assay

Cell proliferation was determined using the CellTiter-Glo Luminescent assay (Promega). Briefly, PDAC cells were seeded in 96-well plates at 2000/well overnight and then were serum starved and incubated with PD-L1 (1–10 μg/ml in 0.1% BSA in PBS) for 24 h. In some experiments cells underwent null treatment with 0.1% BSA as control. Cell proliferation was determined by measuring luminescence using the Spectramax microplate reader.

### Creation of PD-1 knockdown PANC-1 cells

PD-L1 expression on PDAC cells was previously reported;^16–18^ therefore, we chose to further investigate and to knockdown *PD-1* expression, which is primarily expressed on immune cells and has not been characterized on PDAC cells. Three constructs of lentiviral short-hairpin RNA (shRNA) against human *PD-1* (*PDCD1*) (NM_005018) or scramble shRNA-control (Genecopoeia) with the mCherry reporter gene were transfected into PANC-1 cells using jetPRIME reagent (Polyplus) and were selected with puromycin (ThermoFisher). *PD-1* knockdown efficiency was assessed by western blot assay and the most efficient *PD-1* shRNA was chosen. Stably transfected PANC-1 cells were further flow-sorted for >95% purity. PANC-1 *PD-1* knockdown cells along with PANC-1 cells transfected with scramble shRNA were used for cell signaling and xenograft assays.

### PD-1/PD-L1 axis activation of mitogen-activated protein kinase signaling

PDAC cells were plated in 6-well plates at 5 × 10^5^/well and incubated overnight. Cells were starved for 4 h and treated with PD-L1 (1 µg/ml) for 5, 10, 15, 30, and 60 min. Since prior reports have shown that immune checkpoints activate the mitogen-activated protein kinase (MAPK) pathway in immune cells, we sought to determine whether MAPK was activated in PDAC cells by PD-1/PD-L1 signaling.

Cell lysates were collected and probed with anti-phospho and anti-total ERK (Cell Signalling). For blocking assays, cells were pretreated with pembrolizumab (100 µg/ml) for 30 min prior to treatment with PD-L1. To further verify that the PD-1/PD-L1 interaction activated signaling pathways, we repeated treatment assays using PANC-1 cells with *PD-1* knockdown.

### Pancreatic cancer cell lines and organoid cytotoxicity assays

To test whether ICIs were directly cytotoxic to PDAC cells, cultured MIAPaCa-2 and PANC-1 cells were exposed to nivolumab, pembrolizumab, atezolizumab, and IgG antibody controls (trastuzumab and daratumumab). Direct cytotoxicity and combination therapy with the small molecule trametinib (anti-MEK1/2) was also assessed in PDOs, which were developed as previously described.^10,19^ All of the above drugs were selected because they are FDA approved and are used in current clinical practice.

In brief, MIAPaCa-2 and PANC-1 cells were seeded in 96-well plates at 5 × 10^3^ cells/well and exposed to drugs at 1 mg/ml on the second day for 48 h.^20,21^ To measure cytotoxicity in PDOs, organoids were passaged and suspended in BME and seeded in 48-well plates (20 µl/well), designated as day 0. Antibodies and trametinib were added on days 1 and 3; photomicrographs of each treatment group were taken, and cell viability assays were also performed on day 5.^22^ Cytotoxic effects were measured using CellTiter-Glo luminescent assay (Promega) and luminescence was measured using the Spectramax microplate reader.

### Consents and approvals

PDAC tissues were obtained from patients undergoing curative intent surgical resection at Stony Brook University Hospital. Institutional Review Board approval was obtained for tissue acquisition and analysis. Patients provided written informed consent for research analysis of their tissues. Fresh, room temperature PDACs were provided to research personnel following removal from patients.

### Creation of pancreatic cancer xenograft animals

Stony Brook University Institutional Animal Care and Use Committee approved the animal studies, which utilized 6–12-week old NSG mice (The Jackson Laboratory). To create PDTXs, we utilized a standard operating procedure to implant tissues into mice within 30 min of surgical excision.^23^ In brief, PDACs were removed en bloc in the operating room, taken to pathology, and then distributed by a surgical pathologist to provide portions for PDO and PDTX development. For PDTX, tissues (20–30 mm^3^) were implanted subcutaneously into both left and right flanks of mice designating passage 0. About 2–4 months later with positive tumor growth, tumor tissues were harvested and split into three mice denoting passage 1. Thereafter, growing tumors were further expanded into mice designating passage 2 for drug treatment studies.

### Creation of radio-immunoconjugates and positron emission tomography scans

Radio-immunoconjugates of pembrolizumab (^89^Zr-DFO-pembrolizumab) were created using standardized methods.^24^ In brief, pembrolizumab was conjugated with the chelating agent deferoxamine (DFO) and then labeled with the radio-isotope ^89^Zr. With this radio-immunoconjugate, pembrolizumab binds with high affinity to PD-1 and ^89^Zr is detected by positron emission tomography (PET) scan. For in vivo evaluation, PDTXs underwent intraperitoneal injection of ^89^Zr-DFO-pembrolizumab. As a control, PDTXs were injected first with unlabeled pembrolizumab prior to injection with ^89^Zr-DFO-pembrolizumab. Imaging was performed on the Inveon micro-PET/CT scanner (Siemens) to image and quantify radio-immunoconjugate uptake (i.e., PD-1 expression).

### Patient-derived tumor xenograft drug treatment

One month after ensuring tumor growth, PDTX mice were randomly divided into treatment groups (*N* = 3 mice per group) for a 3–4-week period: Group 1, no treatment/control; Group 2, pembrolizumab (25 mg/kg, iv biweekly); Group 3, trametinib (1 mg/kg, po daily); Group 4, pembrolizumab + trametinib.^25^ Pembrolizumab was selected for in vivo assessment because it demonstrated the highest levels of cytotoxicity in PDAC cells and PDOs. Tumor size was measured twice weekly and relative tumor volumes were compared between treatment groups, where relative tumor volume equaled the tumor volume at study time points relative to the initial tumor volume. The cytotoxicity of the treatment arms was evaluated with six PDTXs per treatment arm that was evaluated in two separate studies.

Xenograft animals were also created by implantation of PANC-1 cells that were stably transfected with *PD-1* or scramble shRNA. PANC-1 cells (1 × 10^6^) in 100 µl PBS with 50% BME (R&D) were subcutaneously injected into flank regions of NOD/SCID mice (*N* = 3 animals for each of the two study arms). Tumor size was measured twice weekly over the 6-week study period using calipers; and tumor volume was calculated using the formula *V* = ½ (length × width^2^).^26^

### Statistical analysis

The results presented are representative of three independent experiments run in triplicate, unless otherwise indicated. Statistical tests were performed using GraphPad Prism 5.0 software (GraphPad Software Inc.). For two-group analysis, a two-tailed Student’s *t* test was used to examine group differences. One-way ANOVA with post hoc Tukey’s test was used for multigroup comparison. For the PDTX treatment, the results from the two separate drug studies were combined to provide changes in tumor volume. Differences were considered significant at *P* < 0.05. Results are expressed as mean ± standard error.

## Results

### PD-1 expression in pancreatic cancer cells

**Fig. 1:**
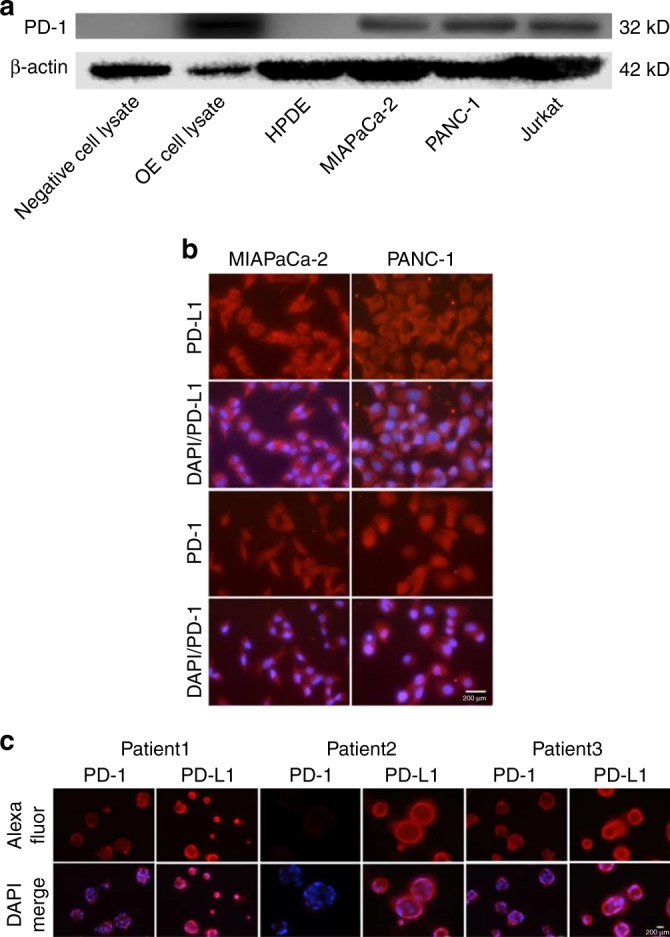
Immune checkpoint expression on PDACs. **a** Western blot assay revealed positive immunostaining for PD-1 in MIAPaCa-2 and PANC-1 cells. Jurkat cells and PD-1 overexpression cell lysate were used as positive controls. Negative control was empty vector cell lysate. ß-actin was used for loading control. **b** Immunofluorescent staining was performed for PD-1 and PD-L1 expression in MIAPaCa-2 and PANC-1 cells. Positive PD-1 and PD-L1 immunostaining was observed in both cell lines. Merged images with DAPI nuclear stains were also constructed for both PD-1 and PD-L1. **c** Immunofluorescence was performed to assess expression of PD-1 and PD-L1 in 3 PDAC PDOs. PD-1 and PD-L1 immunostaining was observed for both patients 1 and 3. However, PD-1 immunostaining was absent in PDOs from patient 2. The second row was a merge with DAPI nuclear staining

Western blot assay demonstrated positive PD-1 protein expression for both PDAC lines and for Jurkat cells (positive cell line control)^27^ (Fig. 1a). IF for PD-1 and PD-L1 were performed and also demonstrated expression in both PDAC cells (Fig. 1b). Controls with secondary antibodies alone for IF demonstrated absence of immunostaining in the two PDAC lines (data not shown). Together, these two assays showed positive PD-1 protein expression in both PDAC lines.

Next, we assessed PD-1 and PD-L1 expression by IF in three PDOs. First, we verified pancreatic epithelial origin of PDOs by showing positive immunostaining for keratin19 (K19) and Sox9 (Supplementary Fig. 1).^10,28^ Then, as shown in Fig. 1c, we observed expression of PD-1 and PD-L1 in 2 PDO lines (patients #1 and #3), whereas the third PDO line (patient #2) had PD-L1 expression alone. Controls with secondary antibodies alone demonstrated absence of immunostaining for both PD-1 and PD-L1 in all three PDOs (data not shown). Collectively, these data demonstrated positive PD-1 expression on PDAC cell lines and PDOs.

### PD-1 expression in human datasets

**Fig. 2:**
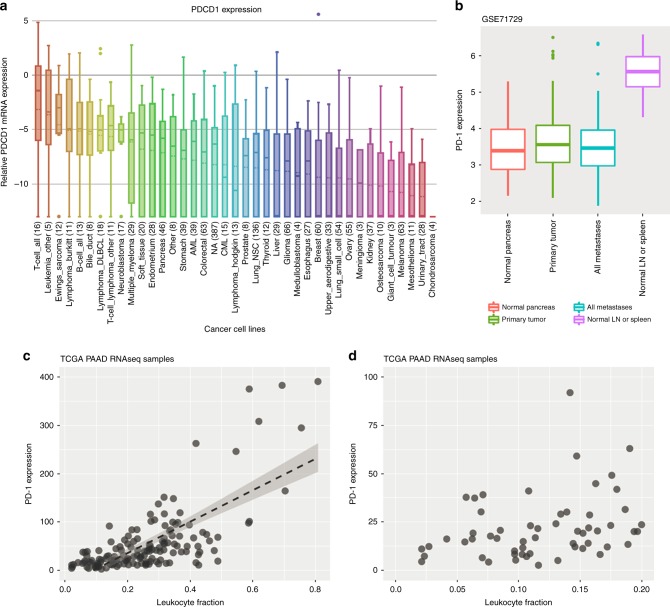
**a** The Broad Institute cancer cell line database was queried for RNASeq analysis of *PD-1* (*PDCD1*) expression levels in hundreds of cancer cell lines. Pancreatic cancer cells (*N* = 46) had PD-1 expression levels higher than most solid organ cancer cell lines, including lung, kidney, colorectal, liver, and melanoma. **b** Gene expression analysis of the GSE71729 clinical PDAC dataset with primary (*N* = 145) and metastatic (*N* = 61) PDAC tumors; and adjacent normal pancreas (*N* = 46) and distant location normal samples (*N* = 88). Query for *PD-1* gene expression revealed similar expression levels between tissues from normal pancreas, primary PDACs, and metastatic PDACs. Normal lymph nodes (LN) or spleen tissues had the highest levels of *PD-1* expression, which is consistent with higher expression levels in immune cells. **c** The TCGA PDAC dataset (*n* = 150) was queried for *PD-1* gene expression which was plotted in relation to the estimated fraction of leukocytes or white blood cells. This analysis revealed that *PD-1* expression directly correlated with the leukocyte fraction. **d** However, inset enlargement of the region of Fig. 2c with low-leukocyte fraction (0.0–0.2) revealed that primary PDACs express *PD-1* even when infiltrating leukocyte populations are low

To assess the relative levels of *PD-1* gene expression in large numbers of PDAC cell lines and clinical specimens, we obtained next generation sequencing data from a public dataset (the Broad Institute) and two prior studies of human PDACs.^7–9^ Query of the Broad Institute RNASeq profiling of cancer cell lines demonstrates that *PDCD1* (i.e., *PD-1*) gene expression levels for PDAC cells were among the highest for solid organ cancers and notably higher than *PDCD1* expression levels in cancers that are routinely treated with ICIs (e.g., colorectal cancer, lung cancer, and melanoma) (Fig. 2a).

When analyzing GSE71729, samples from normal spleen or lymph nodes had the highest levels of *PD-1* expression which is consistent with the higher numbers of immune cells in these tissues. *PD-1* gene expression was lower but distinctly positive in primary and metastatic PDAC tissues. Notably, normal pancreatic tissues also expressed *PD-1* (Fig. 2b). Data from TCGA revealed that *PD-1* gene expression in primary PDACs correlated with the leukocyte fraction (Fig. 2c). However, TCGA data also revealed clearly positive *PD-1* gene expression in primary PDACs even with minimal leukocyte infiltration (Fig. 2d). These cell line and clinical datasets further demonstrate that human PDACs autonomously express *PD-1*.

### The PD-1/PD-L1 axis enhances pancreatic cancer proliferation

**Fig. 3:**
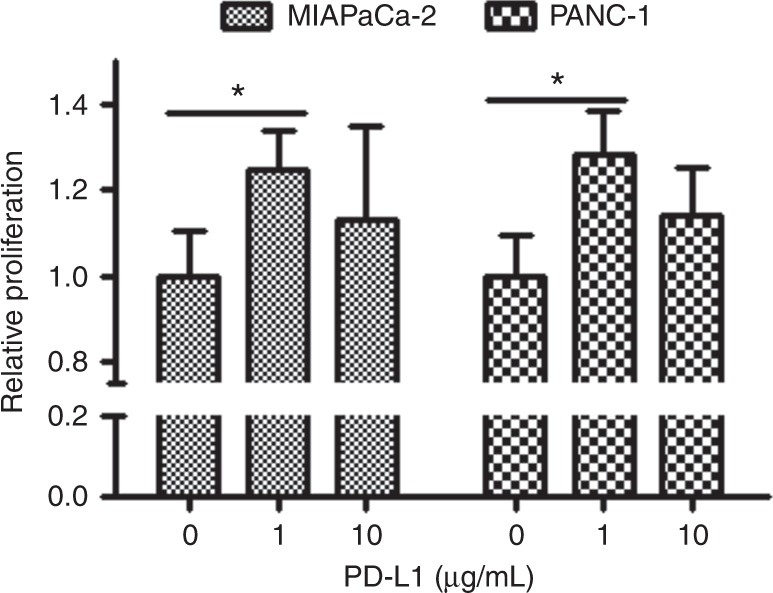
Cell proliferation assays were performed to assess whether PD-1/PD-L1 signaling enhanced growth in MIAPaCa-2 and PANC-1 cells. Serum-starved cells were exposed to recombinant PD-L1 in 0.1% BSA and proliferation was measured after 24 h. Significant increases in cell proliferation were observed in both MIAPaCa-2 and PANC-1 cells (^*^P < 0.05). Null treatment consisted of 0.1% BSA alone

Having demonstrated both PD-1 protein expression and *PD-1* gene expression in PDAC lines, the effects of PD-1/PD-L1 signaling on PDAC growth was determined. Using the CellTiter-Glo luminescent assay, PD-L1 was noted to significantly enhance PDAC cell proliferation (Fig. 3).

### The PD-1/PD-L1 axis activates the mitogen-activated protein kinase pathway

**Fig. 4:**
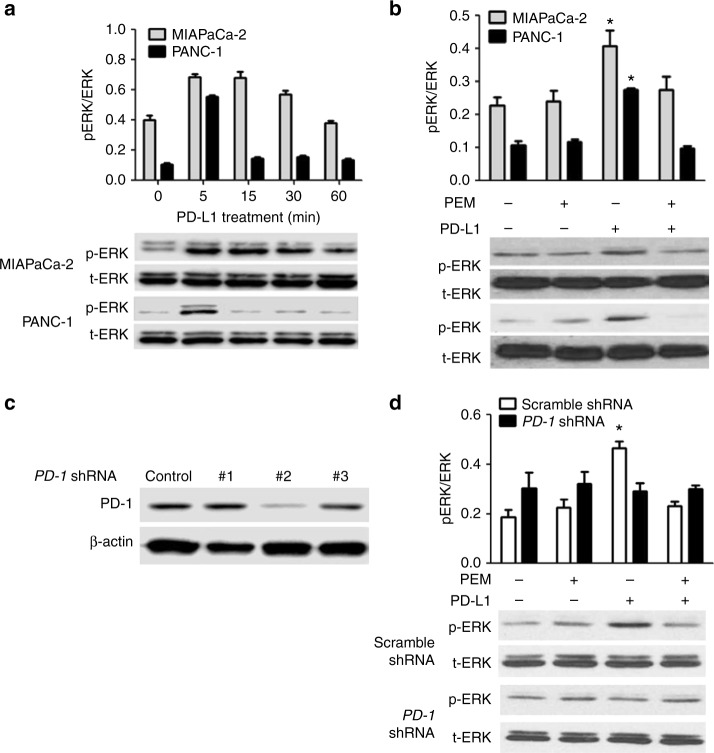
**a** Western blot assay was performed to assess changes in ERK phosphorylation. MIAPaCa-2 and PANC-1 cells were serum starved for 4 h (time point, 0 min) and then exposed to recombinant PD-L1 (1 µg/ml) for 5–60 min. In both cell lines, exposure to PD-L1 increased phospho-ERK. Increases in ERK phosphorylation were quantified by densitometry analysis. **b** Pembrolizumab (anti-PD-1) effectively blocked PD-L1-mediated ERK phosphorylation in both PDAC cell lines. Densitometry verified that ERK phosphorylation with PD-L1 treatment was blocked by pembrolizumab (^*^P < 0.05 compared to null treatment). **c** To further test whether ERK phosphorylation was directly dependent on the interaction between PD-L1 and PD-1, we created stably transfected *PD-1* knockdown PANC-1 cells. Western blot assay revealed that *PD-1* knockdown was most efficient with shRNA2. ß-actin was used as loading control. **d** In *PD-1* shRNA2-transfected PANC-1 cells, we observed that ERK phosphorylation was attenuated following exposure to PD-L1. In contrast, ERK phosphorylation was not blocked in cells with scramble shRNA. Total ERK was used as loading control. Densitometry revealed significantly increased ERK phosphorylation with PD-L1 treatment only in PANC-1 cells with scramble shRNA (^*^P < 0.01)

Since we observed that PD-1/PD-L1 signaling increased cell proliferation, we sought to determine whether it activated oncogenic signaling pathways in PDAC cells. Based upon prior observations with immune checkpoint activation of MAPK and PI-3K/AKT pathways in T cells,^29^ we assessed these pathways here. Following treatment of cultured PDAC cells, we observed that exposure to PD-L1 increased phosphorylation of ERK in both cell lines (Fig. 4a), but did not change phosphorylation levels of AKT in either cell line (data not shown). We also pretreated both PDAC lines with pembrolizumab (anti-PD-1 monoclonal antibody) and observed inhibition of ERK phosphorylation when cells were exposed to PD-L1 (Fig. 4b). Densitometry analysis of the blots quantified the changes in ERK phosphorylation (Fig. 4a, b).

To show that activation of MAPK signaling was specific to the PD-1/PD-L1 interaction, we used a lentiviral vector to stably transfect *PD-1* shRNAs into PDAC cells. We selected PANC-1 cells because these cells had higher levels of PD-1 protein expression by western blot assay and IF. We assessed the efficacy of *PD-1* knockdown with 3 different shRNAs and selected shRNA2 for subsequent studies (Fig. 4c). Successful transfection of lentiviral vectors with *PD-1* shRNA2 and scramble shRNA into PANC-1 cells was verified by mCherry fluorescence (Supplementary Fig. 2). *PD-1* knockdown cells were isolated with puromycin and then exposed to recombinant PD-L1, demonstrating attenuation of ERK phosphorylation (Fig. 4d). Altogether, these assays indicate that PD-1 selectively interacts with PD-L1 to activate MAPK signaling in PDAC cells.

### PD-1 expression enhances pancreatic cancer growth in vivo

**Fig. 5:**
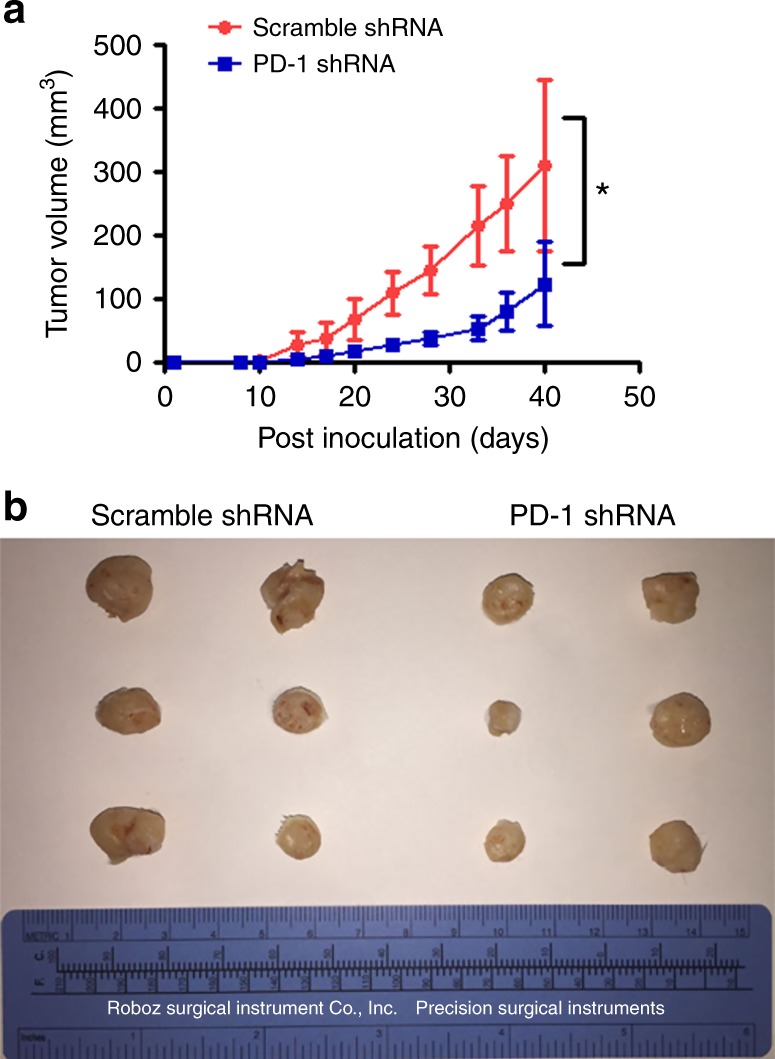
Role of PD-1 expression in PDAC growth. PANC-1 cells (1 × 10^6^) with scramble shRNA or *PD-1* shRNA were implanted into the right and left flank regions of NOD/SCID mice (*n* = 3 animals for both study arms). Tumor volumes were measured twice weekly by calipers. The volumes of the two flank tumors in each animal were averaged; and then tumor volumes were averaged for the three animals in each study group. **a** By day 12, the scramble shRNA tumors exhibited accelerated rates of tumor growth compared to *PD-1* shRNA tumors. By the end of the study period at 40 days, there was approximately 67% smaller tumor volumes in the *PD-1* shRNA tumors compared to scramble shRNA tumors (^*^P < 0.05). **b** At necropsy, the tumors were excised from the mice and the scramble shRNA tumors demonstrated grossly larger tumor volumes compared to *PD-1* shRNA tumors

Since the PD-1/PD-L1 axis enhanced cell proliferation and activated the MAPK pathway in vitro, we sought to determine whether PDAC growth in vivo was also dependent on PD-1 expression. Our results from xenotransplantation of PANC-1 cells with stably transfected *PD-1* shRNA2 into NOD/SCID mice showed significantly smaller tumor volumes in *PD-1* knockdown tumors (*N* = 3 mice, *N* = 6 tumors) compared to scramble/control shRNA tumors (*N* = 3 mice, *N* = 6 tumors) (*P* < 0.01) (Fig. 5a). At the end of the study period, *PD-1* knockdown tumors exhibited approximately 67% smaller tumor volumes compared to scramble shRNA tumors (Fig. 5a, b). The red fluorescent protein mCherry was detected in both *PD-1* shRNA2 and scramble shRNA tumors by in vivo imaging (Xenogen IVIS^®^ Lumina) at the end of the study period, thus verifying the continued presence of successfully transfected lentiviral vectors (Supplementary Fig. 3).

### Radio-immunoconjugate uptake by positron emission tomography

To further assess PD-1 expression levels and selective uptake of anti-PD-1 drugs in PDAC tissues, we utilized PDTXs and radio-immunoconjugates. After successful PDAC implantation and growth in PDTXs, ^89^Zr-DFO-pembrolizumab (64 μCi in 100 μl) was administered by intraperitoneal injection into PDTXs. For control, one animal received an equal concentration of unlabeled pembrolizumab immediately prior to injection with ^89^Zr-DFO-pembrolizumab. Imaging of the animals demonstrated discrete radioactive uptake in the PDAC tissues, indicating positive detection of PD-1 expressing cells (Supplementary Fig. 4).

### PD-1 is a therapeutic target on pancreatic cancer cells

**Fig. 6:**
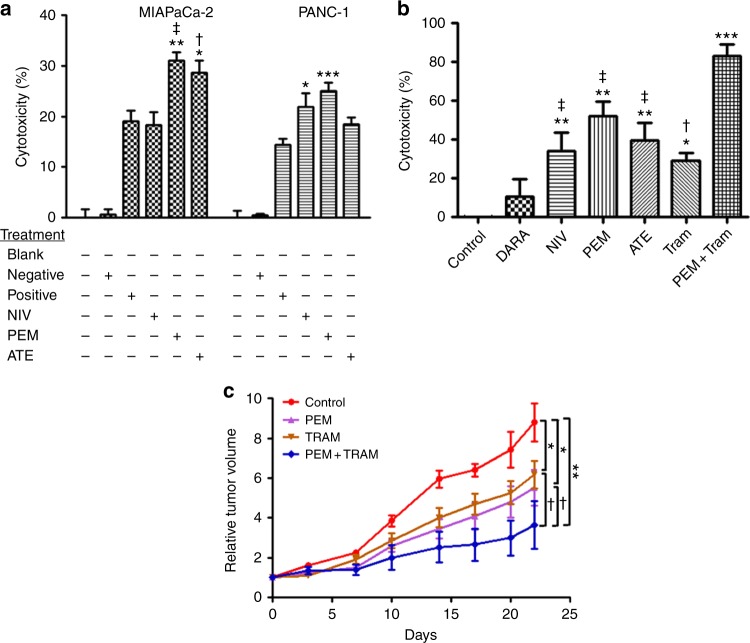
Checkpoint inhibitor cytotoxicity in cell lines, organoids, and xenografts. The cytotoxic effects of pembrolizumab (PEM), nivolumab (NIV), and atezolizumab (ATE) were assessed in MIAPaCa-2 and PANC-1 cells. **a** Treatment arms included blank (no treatment) and two IgG antibody controls (negative target, anti-CD38; and positive target, anti-HER2) for 48 h. Cytotoxicity was not observed or negligible with blank and IgG negative target controls in both cell lines. IgG control with positive target resulted in 15–20% cytotoxicity in the two cell lines. Overall, cytotoxic effects were highest and most consistent for PEM (^**^P < 0.01, ^***^P < 0.001, compared to controls; ^†^*P* < 0.05 and ^‡^*P* < 0.01, compared to NIV). **b** The cytotoxic effects of NIV, PEM, and ATE were assessed in PDOs. Treatment arms also included control (no treatment) and IgG negative target control (daratumumab, DARA). Trametinib (TRAM) was combined with PEM, which exhibited highest cytotoxicity in PDAC cells. PDOs in triplicate were exposed to drugs on day 1 and day 3 and cytotoxicity was measured on day 5 by CellTiter-Glo assay. Results showed 30–50% organoid death with NIV, PEM, and ATE alone; and TRAM alone also yielded approximately 25% organoid death (^*^P < 0.05, ^**^P < 0.01, ^***^P < 0.001, compared with negative control group). When TRAM and PEM were combined, PDO cytotoxicity was greater than 80% (^†^*P* < 0.05 and ^‡^*P* < 0.01, compared to PEM + TRAM). **c** PDTXs were categorized into control group (null treatment) and three different treatment arms (PEM, TRAM, and PEM + TRAM). Animals were treated during the second passage of tumors (*N* = 6 per treatment group). Animals in the control group had significantly larger relative tumor volumes than animals in the study groups (^*^P < 0.05, ^**^P < 0.01 compared with control group). Relative tumor volumes in the PEM + TRAM group were significantly smaller than TRAM, PEM, and controls (^†^*P* < 0.05 compared to PEM + TRAM). The results of two independent treatment groups were combined

We exposed PDAC cell lines to the FDA approved clinical ICIs pembrolizumab, nivolumab, and atezolizumab and observed direct cytotoxicity despite the absence of immune cell infiltrates (Fig. 6a). Our results revealed significantly increased levels of cytotoxicity above null treatment and IgG antibody controls in both cell lines (*P* < 0.01). Next, we assessed the cytotoxicity of these drugs in three PDO lines. Since we identified activation of the MAPK pathway by the PD-1/PD-L1 axis, we sought to determine whether the MEK1/2 inhibitor trametinib could enhance cytotoxicity when combined with pembrolizumab, the ICI with the highest cytotoxic effects in PDAC cells. We observed shrinkage and increased density of organoids with study drug treatment, which have been reported as measures of organoid death.^22^ Minor shrinkage with minimal darkening was observed with the isotype antibody control (Supplementary Fig. 5). By CellTiter-Glo luminescent assay, we also observed significantly increased tumor killing (>80%) with combination therapy (*P* < 0.01, compared to all other treatment arms), whereas ICIs alone yielded levels of cytotoxicity closer to 30–50% (*P* < 0.01, compared to controls) (Fig. 6b).

Finally, we treated PDTXs with ICI alone and in combination with trametinib (*N* = 6 PDTXs for each treatment arm). We selected pembrolizumab for the reasons noted above. All mice remained active and maintained stable body weights (18–22 gm) for the entire treatment period. After treatment, we observed that tumor growth was significantly attenuated with pembrolizumab or trametinib alone compared to controls (*P* < 0.01 and *P* < 0.05, respectively). However, we observed greatest blockage of tumor growth with pembrolizumab combined with trametinib (compared to control, *P* < 0.01; compared to trametinib, *P* < 0.05; or compared to pembrolizumab, *P* < 0.05) (Fig. 6c). Collectively, these in vivo studies provide evidence that ICIs are cytotoxic to PDACs even in immune-deficient cancer models; and that rationally designed drug combinations have the potential to yield considerably higher cancer cell cytotoxicity in human PDACs.

## Discussion

Immune checkpoints have critical roles in maintaining immune homeostasis and preventing autoimmunity, and cancers have exploited these mechanisms to acquire resistance to immune surveillance and to escape death from cytotoxic T cells.^30^ Here, in our investigational studies, we discovered that PDACs may utilize immune checkpoints to enhance cancer growth through novel means. Importantly, we discovered that PDAC cells express PD-1, which has been thought to be primarily expressed only on immune cells.^31,32^ We observed that PD-1 expression contributed to PDAC growth and to activation of oncogenic signaling pathways. Thus, it is reasonable speculation that ICIs could antagonize PDAC growth by blocking these PD-1 protumourigenic pathways. Indeed, our data supports this concept by showing direct cytotoxicity on PDAC cells and tissues when using clinical ICIs in cancer models that are deficient in cytotoxic immune cell populations.

To investigate immune checkpoint function in pancreatic cancer, we investigated the MAPK and PI-3K/AKT pathways and observed increased levels of ERK phosphorylation but not AKT phosphorylation when PDAC cells were exposed to PD-L1. The decision to examine MAPK was based on several factors including prior observations in T cells demonstrating PD-1 regulation of MAPK and PI-3K/AKT pathways,^29^ which are critical signaling pathways in pancreatic cancer pathogenesis and progression. Indeed, activation of MAPK signaling (but not PI-3K/AKT) recapitulated the effects of activated *KRAS* in mice, supporting the role of MAPK as an essential effector of *KRAS* in PDAC.^33,34^ Indeed, our group has also demonstrated MAPK-dependent pancreatic cancer growth;^3,4^ and targeting MAPK should reasonably lead to cytotoxic outcomes. In fact, FDA approved drugs targeting the MAPK pathway are currently available.^35^ It also remains feasible, however, that the PD-1/PD-L1 axis may enhance pancreatic cancer growth by unexplored signaling pathways. As such, we are currently preparing studies to perform a comprehensive analysis of activated pathways. Nevertheless, our studies show the importance of the MAPK pathway as a mediator of PD-1/PD-L1 signaling and as a candidate therapeutic target that may provide increased PDAC death in combination with ICIs.

Our observation that ICI drugs were cytotoxic to PDAC cells and tissues independent of T-cell activity is directly relevant to the clinical management of PDAC patients and are noteworthy on several different points. First, we tested nivolumab, pembrolizumab, and atezolizumab, which are currently in routine clinical use and can be quickly implemented in human clinical trials. Second, our studies revealed variable and considerable levels of cytotoxicity with single agent ICI treatment. Although single agent ICI therapies and nonmechanistic based regimens have been ineffective for human PDACs in clinical trials,^36–38^ our results nevertheless indicate positive drug activity which can be the foundation to develop better, more lethal combination therapies. Indeed, our subsequent studies revealed that combination therapies targeting both immune checkpoints and the MAPK pathway produced the highest levels of cytotoxicity. Such results can strengthen the design of future studies that build upon more recent positive clinical trials evaluating ICIs in human PDACs.^39,40^ Importantly, however, it remains unclear what contribution an intact immune system may have towards further increasing the cytotoxic killing of PDAC cells. Salazar and colleagues demonstrated considerable ICI response in immunocompetent PDAC mice and, thus it is plausible that optimal cytotoxic effects from ICIs in clinical regimens may require both direct PDAC targeting combined with robust immune cell responses.^41^

There are important technical considerations regarding the results of our study. The use of organoids to test the efficacy of drugs remains in its infancy, although a recent report indicates that organoids are accurate predictors of treatment response in patients with gastrointestinal malignancies.^42^ Furthermore, our investigational studies revealed that the cytotoxic effects of our drugs were generally consistent between PDOs and PDTXs. PDOs have the potential to be rapidly and easily developed as cancer models that can provide personalized drug-sensitivity data.^28^ For example, the development of PDOs occurred on a scale measured by weeks compared to months for PDTXs. Thus, drug-sensitivity testing and real-time decisions on drug selection and administration may be feasible with PDOs, but perhaps not possible with PDTXs. This association also carries great importance to clinical medicine because accurate predictors of treatment response remain lacking for many therapies. In fact, there is no accurate biomarker for ICIs, since it has become increasingly clear that optimal treatment responses from ICIs do not directly correspond to immunohistochemical analysis of immune checkpoint expression.^43–47^

Our current studies do not detract from the known mechanisms of adaptive immune response. Instead, our studies reveal that immune checkpoints have intrinsic growth supporting functions in PDAC cells and that ICIs may yield cancer cell death by not only facilitating robust cytotoxic immune responses, but also by direct cytotoxicity on cancer cells. In conclusion, immune responses involving PD-L1 on cancer cells binding with PD-1 on T cells to antagonize cytotoxic T-cell activity is an established paradigm and forms the basis of ICI therapy. Based on our current work, tumor autonomous PD-1 and tumor specific immune checkpoint signaling pathways represent viable and promising therapeutic targets in PDAC.

## Electronic supplementary material

Supplementary Figure Legend

Supplementary Figure 1

Supplementary Figure 2

Supplementary Figure 3

Supplementary Figure 4

Supplementary Figure 5

## Availability of data/materials

The corresponding author has access to all data in the study.
