# Correction: Direct therapeutic targeting of immune checkpoint PD-1 in pancreatic cancer

**DOI:** 10.1038/s41416-020-0879-6

**Published:** 2020-05-11

**Authors:** Mei Gao, Miranda Lin, Richard A. Moffitt, Marcela A. Salazar, Jinha Park, Jeffrey Vacirca, Chuan Huang, Kenneth R. Shroyer, Minsig Choi, Georgios V. Georgakis, Aaron R. Sasson, Mark A. Talamini, Joseph Kim

**Affiliations:** 1 Department of Surgery, University of Kentucky, Lexington, KY USA; 2 Markey Cancer Center, University of Kentucky, Lexington, KY USA; 3 Department of Pathology, State University of New York, Stony Brook, NY USA; 4 Department of Experimental Therapeutics, City of Hope, Duarte, CA USA; 5 Department of Radiology, University of Iowa, Iowa City, IA USA; 6 New York Cancer Specialists, East Setauket, New York, NY USA; 7 Departments of Radiology, State University of New York, Stony Brook, NY USA; 8 Departments of Psychiatry, State University of New York, Stony Brook, NY USA; 9 Departments of Medicine, State University of New York, Stony Brook, NY USA; 10 Departments of Surgery, State University of New York, Stony Brook, NY USA

Correction to: *British Journal of Cancer* (2019) **120**, 88-96; 10.1038/s41416-018-0298-0, published online 31 October 2018

The original version of this article contained an error in Fig. 1b. The third row should have been labelled as “PD-1” instead of “PD-L1”. The correct figure is below.

**Fig. 1:**
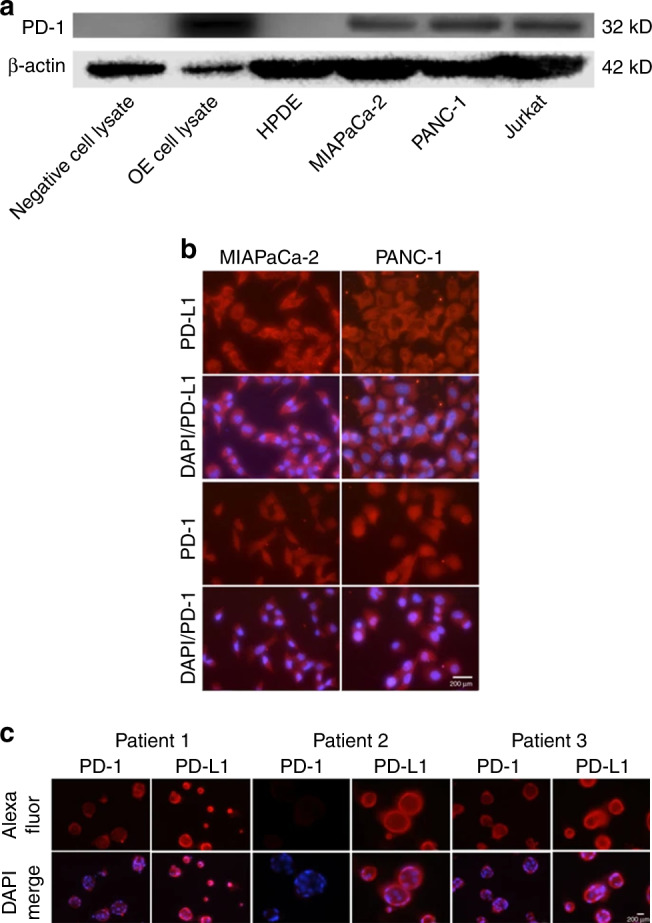
Immune checkpoint expression on PDACs. **a** Western blot assay revealed positive immunostaining for PD-1 in MIAPaCa-2 and PANC-1 cells. Jurkat cells and PD-1 overexpression cell lysate were used as positive controls. Negative control was empty vector cell lysate. ß-actin was used for loading control. **b** Immunofluorescent staining was performed for PD-1 and PD-L1 expression in MIAPaCa-2 and PANC-1 cells. Positive PD-1 and PD-L1 immunostaining was observed in both cell lines. Merged images with DAPI nuclear stains were also constructed for both PD-1 and PD-L1. **c** Immunofluorescence was performed to assess expression of PD-1 and PD-L1 in 3 PDAC PDOs. PD-1 and PD-L1 immunostaining was observed for both patients 1 and 3. However, PD-1 immunostaining was absent in PDOs from patient 2. The second row was a merge with DAPI nuclear staining.

