# Supplementary material for: Direct therapeutic targeting of immune checkpoint PD-1 in pancreatic cancer
Source: Br J Cancer. 2018 Oct 31;120(1):88–96. doi: 10.1038/s41416-018-0298-0 (PMC6325157; doi:10.1038/s41416-018-0298-0)
Supplement: Supplementary file 1 — Supplementary Figure Legend [file 41416_2018_298_MOESM1_ESM.docx]

**SUPPLEMENTARY FIGURE LEGEND**

**Fig. S1.** Immunofluorescence for pancreatic duct markers. Patient derived organoids were assessed for K19 and Sox9 expression by confocal microscopy, demonstrating positive immunostaining for both markers. These results support the pancreatic origin of the organoids. The second row is a merge with DAPI nuclear stains.

**Fig. S2.** Transfection of *PD-1* and scramble shRNA assessed by the Xenogen IVIS® Lumina system. PANC-1 cells were transfected with lentivirus expressing shRNA against *PD-1* and scramble shRNA. The efficiency of transfection was assessed by co-expression of the mCherry reporter gene, which demonstrated red fluorescence as noted above in both *PD-1* (figure, right) and scramble (figure, left) shRNA-transfected PANC-1 cells.

**Fig. S3.** Expression of the red fluorescent protein mCherry in NOD/SCID mice. Lentiviral vectors with shRNA against *PD-1* and scramble shRNA with the mCherry reporter gene were transfected into PANC-1 cells. NOD-SCID animals were inoculated with these PANC-1 cells. At 40 days of tumor growth, NOD/SCID mice continued to demonstrate fluorescence in both scramble and *PD-1* shRNA tumors.

**Fig. S4.** Organoid treatment arms (nivolumab, NIV; pembrolizumab, PEM; atezolizumab, ATE; trametinib, TRAM; and daratumumab, DAR) were imaged prior to cytotoxicity assay. Compared to null treatment, organoids exposed to drugs were smaller in size and appeared denser as they underwent cell death. DAR (IgG control) treated cells also decreased in size, but the organoids generally did not appear more condensed.
