# Supplementary figures and images for: Direct therapeutic targeting of immune checkpoint PD-1 in pancreatic cancer
Source: Br J Cancer. 2018 Oct 31;120(1):88–96. doi: 10.1038/s41416-018-0298-0 (PMC6325157; doi:10.1038/s41416-018-0298-0)

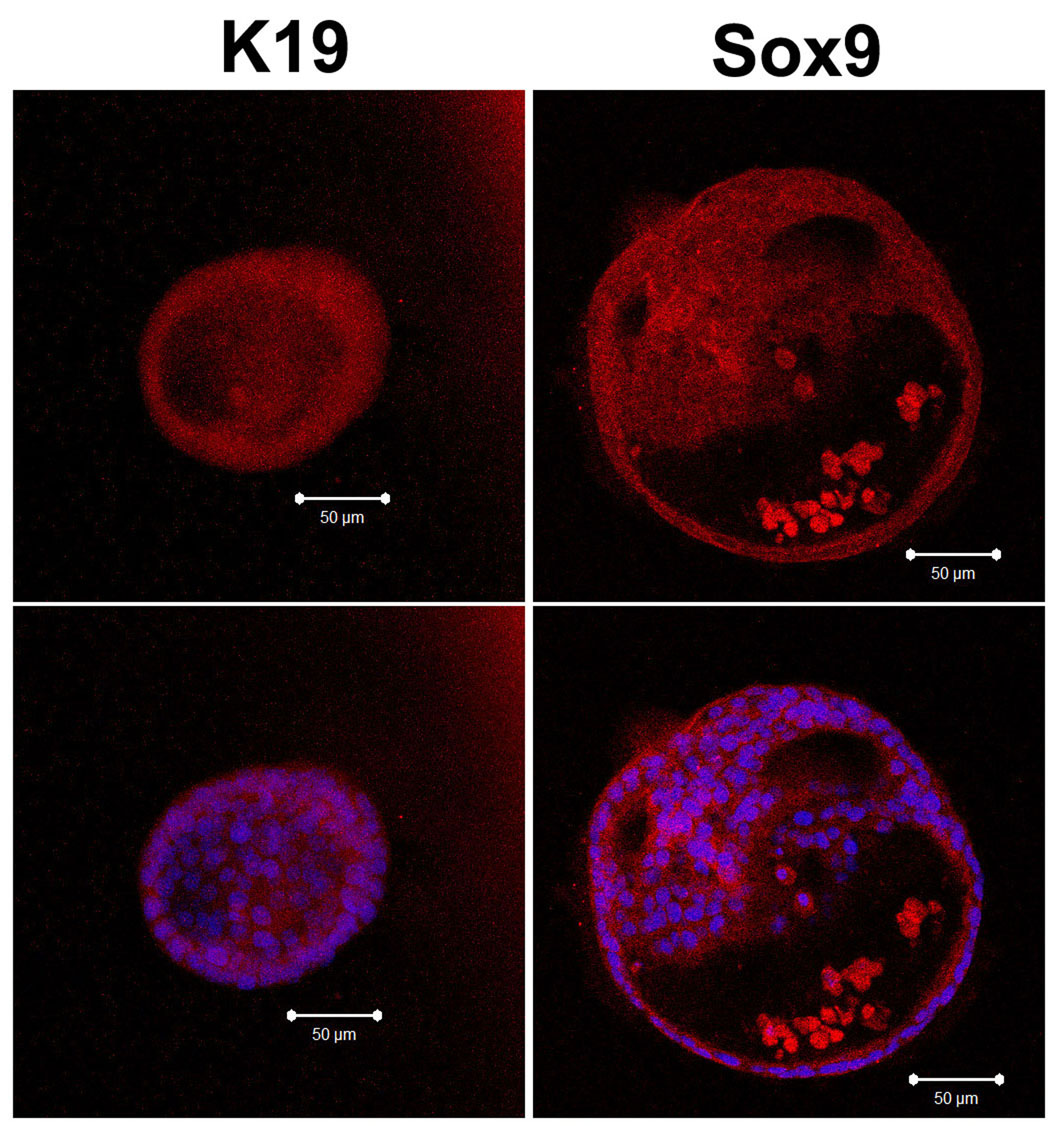

Supplement: Supplementary file 2 — Supplementary Figure 1 [file 41416_2018_298_MOESM2_ESM.jpg]

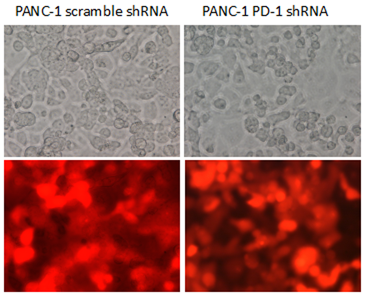

Supplement: Supplementary file 3 — Supplementary Figure 2 [file 41416_2018_298_MOESM3_ESM.tif]

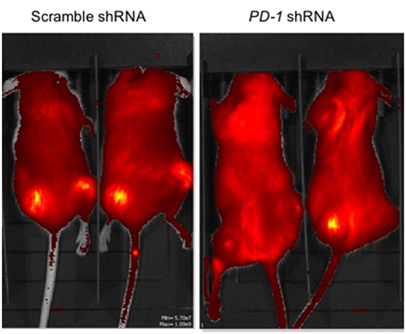

Supplement: Supplementary file 4 — Supplementary Figure 3 [file 41416_2018_298_MOESM4_ESM.tif]

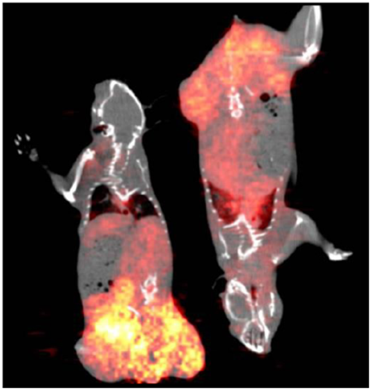

Supplement: Supplementary file 5 — Supplementary Figure 4 [file 41416_2018_298_MOESM5_ESM.tif]

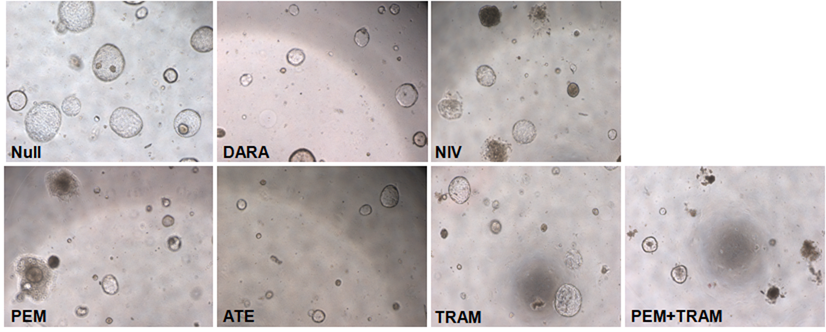

Supplement: Supplementary file 6 — Supplementary Figure 5 [file 41416_2018_298_MOESM6_ESM.tif]
